# Dynamic role of the codon 72 *p53* single-nucleotide polymorphism in mammary tumorigenesis in a humanized mouse model

**DOI:** 10.1038/s41388-018-0630-4

**Published:** 2019-01-16

**Authors:** Ramesh T. Gunaratna, Andres Santos, Linjie Luo, Chandandeep Nagi, Isabel Lambertz, Madison Spier, Claudio J. Conti, Robin S. Fuchs-Young

**Affiliations:** 1 Interdisciplinary Program in Genetics, Texas A&M University, College Station, TX USA; 2 Department of Molecular and Cellular Medicine, College of Medicine, Texas A&M Health Science Center, College Station, TX USA; 3 Department of Molecular Biology, Princeton University, Princeton, NJ USA; 4 Paul L. Foster School of Medicine, Texas Tech University Health Science Center, El Paso, TX USA; 5 Department of Pathology and Immunology, Baylor College of Medicine, Houston, TX USA; 6 Departamento de Bioingeniería, Universidad Carlos III de Madrid, Madrid, Spain; 7 Fundación Instituto de Investigación Sanitaria de la Fundación Jiménez Díaz (IIS-FJD), Madrid, Spain; 8 Centro de Investigación Biomédica en Red en Enfermedades Raras (CIBERER-ISCIII), Madrid, Spain

**Keywords:** Predictive markers, Cancer genetics, Breast cancer

## Abstract

Female breast cancer (BrCa) is the most common noncutaneous cancer among women in the United States. Human epidemiological studies reveal that a *p53* single-nucleotide polymorphism (SNP) at codon 72, encoding proline (P72) or arginine (R72), is associated with differential risk of several cancers, including BrCa. However, the molecular mechanisms by which these variants affect mammary tumorigenesis remain unresolved. To investigate the effects of this polymorphism on susceptibility to mammary cancer, we used a humanized *p53* mouse model, homozygous for either P72 or R72. Our studies revealed that R72 mice had a significantly higher mammary tumor incidence and reduced latency in both DMBA-induced and MMTV*-Erbb2/Neu* mouse mammary tumor models compared to P72 mice. Analyses showed that susceptible mammary glands from E-R72 (R72 x MMTV*-Erbb2/Neu*) mice developed a senescence-associated secretory phenotype (SASP) with influx of proinflammatory macrophages, ultimately resulting in chronic, protumorigenic inflammation. Mammary tumors arising in E-R72 mice also had an increased influx of tumor-associated macrophages, contributing to angiogenesis and elevated tumor growth rates. These results demonstrate that the *p53* R72 variant increased susceptibility to mammary tumorigenesis through chronic inflammation.

## Introduction

Female breast cancer (BrCa) affects more than 2.2 million women each year worldwide [1]. Etiology of BrCa is heterogeneous and involves genetic, environmental, and lifestyle factors. Only 5–10% of BrCas cases are hereditary [2], indicating that the majority are due to genetic susceptibilities affecting response to environmental exposures. Several established risk factors, including obesity and aging, are associated with increased levels of protumorigenic, chronic tissue inflammation [3–5].

The tumor suppressor p53 is the most commonly mutated gene in BrCa, and functional disruption of p53 is associated with increased tumor aggressiveness, refractoriness to treatment and poorer prognosis [6, 7]. P53 regulates cell-autonomous biological activities that maintain genomic integrity, particularly in response to insults such as DNA damage, oncogene activation, and oxidative stress. In response to these stressors, p53 transactivates genes that regulate apoptosis, cell cycle arrest and senescence, facilitating repair or elimination of irreparably damaged cells [8]. However, recent evidence indicates that the anti-cancer effects of p53 can be undermined when persistent cell cycle arrest and accumulation of senescent cells leads to the acquisition of a senescence-associated secretory phenotype (SASP). SASP is characterized by synthesis and secretion of a variety of proinflammatory cytokines and chemokines resulting in chronic inflammation that is protumorigenic [9].

A common single-nucleotide polymorphism of *p53* at codon 72 modifies critical biological processes that impact susceptibility to several cancers. This SNP encodes either proline (P72) or arginine (R72), and is located in the polyproline region of exon 4, between the transactivation and DNA binding domains [10, 11]. In vitro studies with human cancer cells and rodent fibroblasts show that following DNA damage, the P72 variant preferentially promotes cell cycle arrest [12], senescence [13], and DNA repair [14], while the R72 variant more effectively induces apoptosis [15–17].

In vivo analyses in Hupki (Human *p53* exon 4–9 knock-in) mice have shown that these codon 72 variants induce apoptosis and senescence in a tissue and context-dependent manner [18, 19]. Exposure to radiation significantly induces levels of apoptosis in the small intestine of R72 compared to P72 mice. However, P72 animals have significantly higher levels of apoptosis in the thymus [18]. When Hupki mice are fed an obesogenic, high-fat diet, the codon 72 variants differentially regulate genes involved in cellular metabolism and inflammation [19].

Human epidemiological studies of cancer risk have shown modest, but significant associations of codon 72 *p53* variants with incidence in several types of cancers, including lung, prostate, and breast [20–24]. Similar to the Hupki mouse, codon 72 *p53* variants display cell- and tissue-specific activities in humans, and are not associated with increased risk for all cancer types [11].

The mechanisms by which codon 72 *p53* variants differentially affect breast cancer susceptibility remain unresolved. Therefore, we explored this question in a physiologically relevant in vivo setting, using a well-characterized, humanized *p53* exon 4 knock-in mouse model, expressing the codon 72 polymorphisms [17]. Our results showed that the R72 variant induced significantly greater levels of chronic mammary tissue inflammation compared to the P72 variant, contributing to enhanced susceptibility to mammary carcinogenesis.

## Results

### Increased mammary tumor incidence and reduced latency in R72 mice

**Fig. 1:**
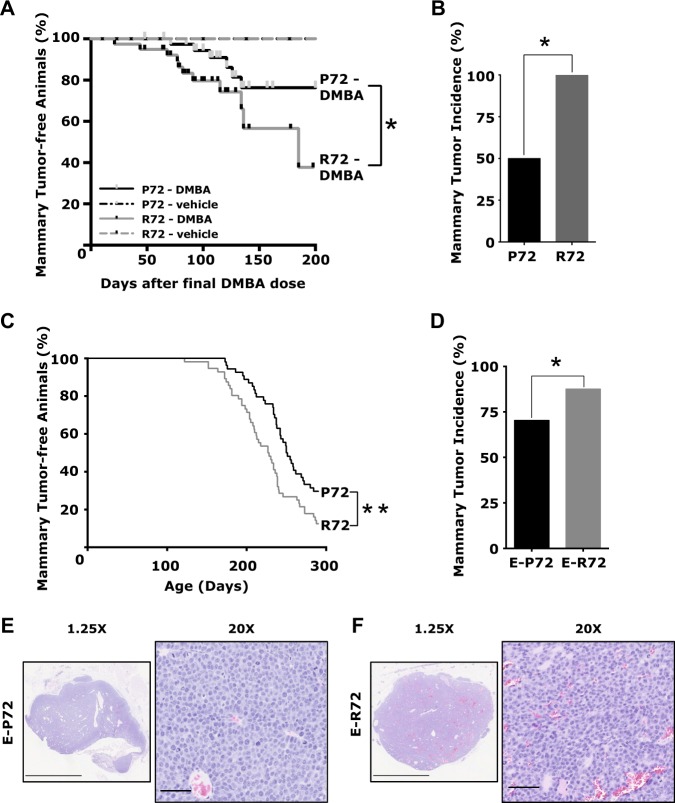
Increased mammary tumor incidence and reduced latency in R72 mice. **A**. Kaplan–Meier survival curves of DMBA or vehicle-treated P72 (*n* = 40) and R72 (*n* = 39) mice (*p < 0.05). **B**. Mammary tumor incidence in DMBA-treated R72 (100%) and P72 (50%) mice (*p < 0.05). **C**. Kaplan–Meier survival curves of bigenic E-P72 (*n* = 54) and E-R72 (*n* = 56) mice (**p < 0.01). **D**. Mammary tumor incidence in E-R72 (87.5%) and E-P72 (70.3%) bigenic mice (*p < 0.05, **p < 0.01). **E**, **F**. Representative H&E images of mammary carcinomas from **E**. E-P72 and **F**. E-R72 bigenic mice. Magnifications 1.25x and 20x, scale bars 2.5 mm and 100 µm, respectively

Initial investigations focused on the impact of the p53 variants at codon 72 on mammary tumor development in two distinct models. First, 7,12-dimethylbenz(a)anthracene (DMBA) was used to induce mammary carcinomas [25]. As shown in Fig. 1A, mice homozygous for the arginine variant, R72 (*n* = 39), had significantly reduced mammary tumor latency compared to those with the proline variant, P72 (*n* = 40, Log-rank test; *p* = 0.049). Mammary tumors first appeared at 21 days post-DMBA in R72 mice, 50 days earlier than in the P72 animals. R72 mice also had a 50% higher mammary tumor incidence compared to P72 animals (Fig. 1B). Mice in the vehicle-treated groups remained tumor-free, indicating that the codon 72 variants did not stimulate spontaneous mammary tumorigenesis in this model (Fig. 1A).

Next, the impact of *p53* polymorphic variants on mammary tumorigenesis was investigated in the MMTV*-Erbb2/Neu* FVB mouse model [26]. Mice were cross-bred to generate study animals that were homozygous for the codon 72 variants and hemizygous for the *MMTV-Erbb2/Neu* transgene (E-P72R). As in the carcinogenesis study, E-R72 mice (*n* = 56) had significantly reduced mammary tumor latency compared to E-P72 (*n* = 54, Log-rank test, *p* = 0.004, Fig. 1C). Mammary tumors first appeared in E-R72 animals as early as 122 days of age, compared to 173 days in E-P72 animals, a 51-day difference. Mean tumor latencies of E-R72 and E-P72 mice were 226 and 250 days, respectively. Mammary tumor incidence was 17% higher in E-R72 animals compared to E-P72 animals (*p* = 0.004, Fig. 1D). As previously observed in *MMTV-Erbb2/Neu* mice, which are on an FVB background [26], mammary tumors in both E-R72 and E-P72 mice were mammary adenocarcinomas with moderate to poor differentiation (Fig. 1E, F). These results show that in comparison to P72, R72 mice had increased mammary tumor incidence and reduced latency in both carcinogenesis and genetic models. Additionally, these findings demonstrate that the *p53* variants altered the susceptibility to tumorigenesis in response to chemical (DMBA) or oncogenic (*Erbb2*) stimuli.

**Fig. 2:**
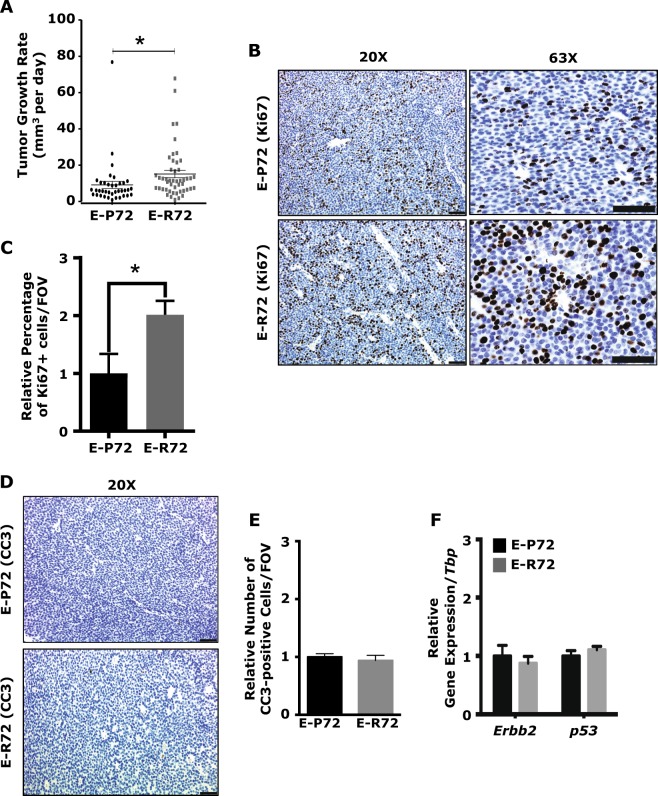
Increased cell proliferation in tumors arising in E-R72 animals. Comparisons are of mammary tumors harvested from E-P72 and E-R72 mice. **A**. Average mammary tumor growth rates (mm^3^/day), in E-P72 (*n* = 38), E-R72 (*n* = 48, *p < 0.05) mice. **B**. Immunolocalization of Ki67. Magnifications 20x and 63x, scale bar, 50 µm. **C**. Percentage Ki67 + cells per high power field of view (FOV, *n* = 5, *p < 0.05). **D**. Representative images of CC3 immunostaining. Magnification 20x, scale bar, 50 µm. **E**. Quantification of CC3 + cells per FOV (*n* = 5, *p < 0.05). **F**. Quantitative RT-PCR analysis (QPCR) of *Erbb2* and *p53*, normalized to *Tbp* (*n* = 4). All values were standardized to the mean of E-P72 samples, and reported as mean ± SEM

These latency results prompted an assessment of tumor progression. Tumors in E-R72 animals had a significantly higher average growth rate (37.8 ± 5.1 mm^3^ per day) compared to tumors in E-P72 mice (22.8 ± 5.0 mm^3^ per day) (Fig. 2A). Immunolocalization of Ki67 revealed significantly higher levels of proliferation in tumors from E-R72 compared to E-P72 mice (Fig. 2B, C). Tumors from both E-R72 and E-P72 mice had comparably low levels of apoptosis (Fig. 2D, E). As shown in Fig. 2F, variation in tumor progression was not due to differential expression of *Erbb2/Neu* and/or *p53*.

### Increased proportion of senescent cells in susceptible mammary glands from E-R72 mice

The local milieu in susceptible tissues can play a critical role in tumor initiation and progression [9, 27]. Previous studies have shown that the codon 72 variants differ in their ability to regulate biological processes that regulate cell survival, and modulate the tissue environment [8, 12–15, 27]. Apoptosis, cell cycle arrest and senescence in the mammary glands of adult E-P72 and E-R72 mice were examined to determine the effect of the codon 72 variants on these processes.

**Fig. 3:**
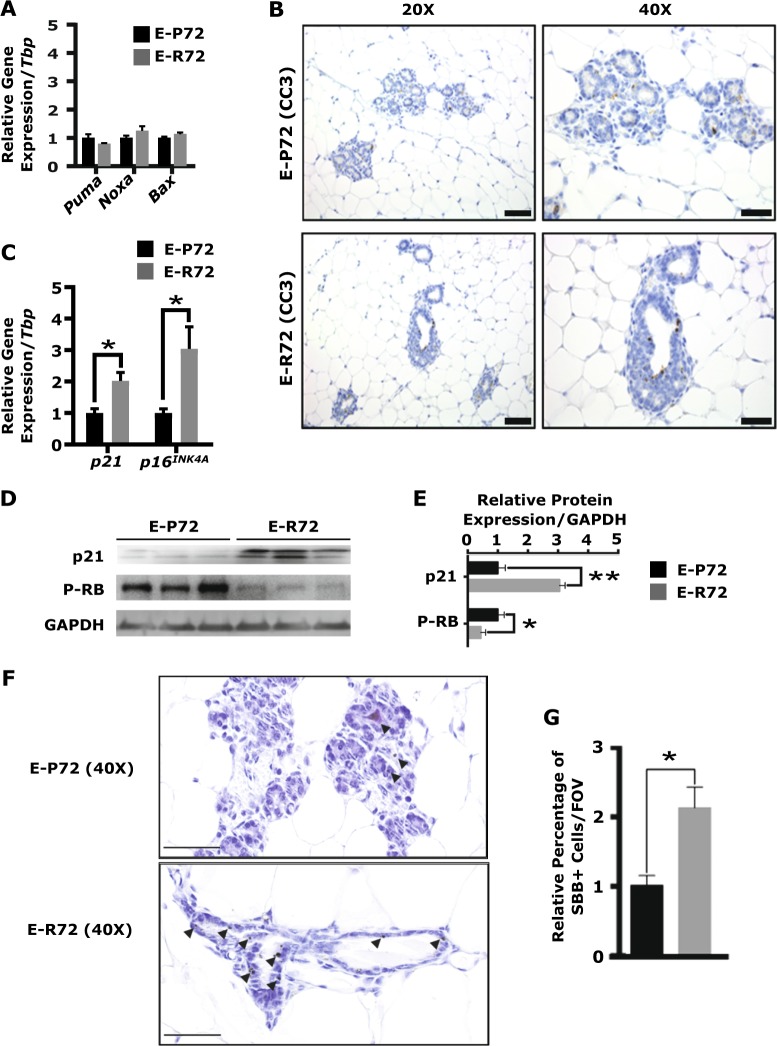
Increased proportion of senescent cells in the susceptible mammary glands from E-R72 mice. Comparisons are of mammary glands harvested from age-matched E-P72 and E-R72 mice. **A**. QPCR analyses of p53-regulated proapoptotic genes normalized to *Tbp* (*n* = 6). **B**. Representative images of CC3 immunostaining in mammary glands. Magnifications 20x and 40x, scale bar, 50 µm. **C**. QPCR analysis of *p21* and *p16*^INK4A^, normalized to *Tbp* (*n* = 6, *p < 0.05). **D**. Representative western blots of p21, phosphorylated RB (P-RB) and GAPDH. **E**. Densitometric quantitation of p21 and P-RB protein, normalized to GAPDH (*n* = 5, **p < 0.01, *p < 0.05). **F**. Sudan Black B (SBB)–positive cells in mammary glands. Arrowheads show SBB+ cells. Magnification 40x, scale bar, 100 µm. **G**. Quantification of the percentage of SBB+ cells per FOV (*n* = 3, *p < 0.05). All values were standardized to the mean of E-P72 samples, and reported as mean ± SEM

First, expression levels of the p53-regulated proapoptotic genes that trigger mitochondrial permeabilization leading to Caspase cleavage were assessed [8]. As shown in Fig. 3A, expression levels of *Puma*, *Noxa*, and *Bax* were similar in mammary glands from E-P72 and E-R72 animals. At the histological level, immunolocalization of CC3 revealed comparably low levels of apoptosis in glands of E-R72 and E-P72 mice (Fig. 3B), indicating that the differences in tumorigenesis were not due to variations in programmed cell death.

Two major effectors of cell cycle arrest and senescence, *p21* and *p16*^INK4a^, mediate their effects by disrupting the formation of Cyclin-CDK complexes and activating RB via hypophosphorylation [28–30]. The active form of RB binds to members of the E2F family, repressing expression of cell cycle progression genes [31]. As shown in Fig. 3C, mRNA expression levels of *p21* and *p16*^INK4a^ were two and three times higher, respectively, in the glands of E-R72 compared to E-P72 animals (*p < 0.05). Mammary glands from E-R72 mice also had highly increased p21 protein expression and significantly decreased levels of phosphorylated RB (P-RB) compared to their E-P72 counterparts (Fig. 3D, E).

Sudan Black B (SBB) identifies senescent cells by binding to lipofuscins, which are aggregates of oxidized proteins and lipids [32]. As shown in Figs. 3F and G, the percentage of SBB+ cells was significantly higher in mammary glands of E-R72 compared to E-P72 animals. These results show that while mammary tumor incidence and proliferation were elevated in E-R72 mice, mammary glands from these animals also had an increased proportion of senescent cells.

### Increased SASP, proinflammatory cytokines, and angiogenic factors in susceptible mammary glands of E-R72 mice

Traditionally, permanent cell cycle arrest, a pre-requisite of cellular senescence, has been thought to inhibit tumorigenesis by preventing expansion of the population of transformed cells. However, recent studies provide strong evidence to the contrary, demonstrating that senescent cells can acquire a senescence-associated secretory phenotype (SASP), which is proinflammatory and protumorigenic. SASP is characterized by secretion of a mixture of proinflammatory cytokines, growth and matrix remodeling factors and chemoattractants that contribute to chronic tissue inflammation [28, 33]. This inflammatory milieu has been shown to stimulate cancer initiation and progression, due to mutagenic and proliferative signals, respectively [34, 35].

**Fig. 4:**
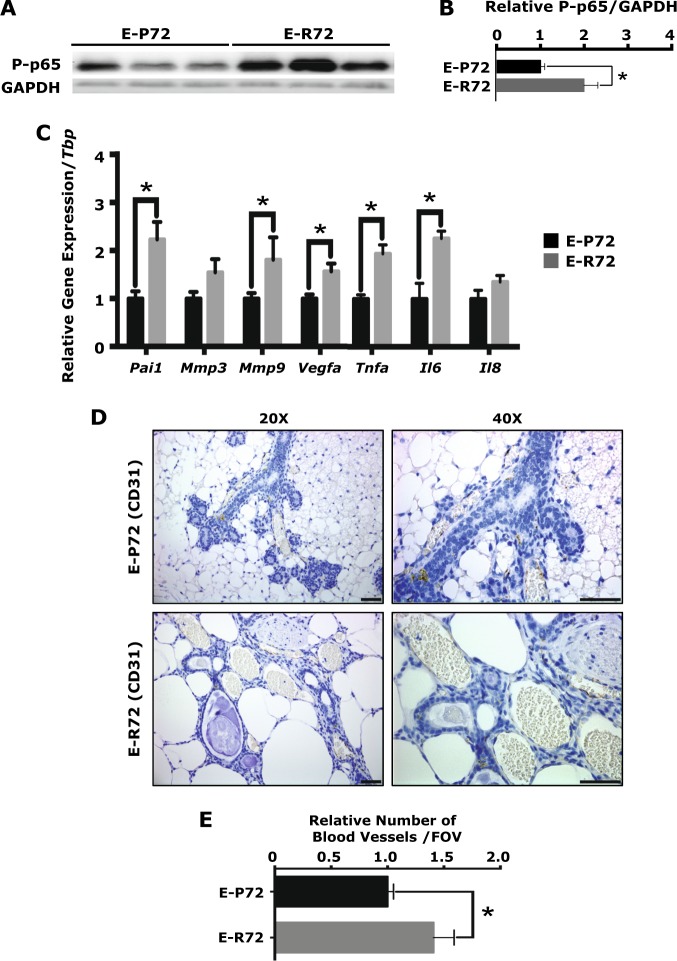
Increased SASP, proinflammatory cytokines, and angiogenic markers in susceptible mammary glands of E-R72 mice. Comparisons are of mammary glands harvested from age-matched E-P72 and E-R72 mice. **A**. Representative western blots of P-p65 (Ser536) and GAPDH. **B**. Densitometric quantitation of P-p65 (Ser536) protein, normalized to GAPDH (*n* = 4, *p < 0.05). **C**. QPCR anaysis of SASP genes normalized to *Tbp* (*n* = 6, *p < 0.05). **D**. CD31 immunolocalization in mammary sections. Magnifications 20x and 40x, scale bar, 50 µm. **E**. Quantification of CD31+ blood vessels per FOV (*n* = 5, *p < 0.05). All values were standardized to the mean of E-P72 samples, and reported as mean ± SEM

RelA (p65), one of five transcription factors in the NFκB signal transduction pathway, is phosphorylated in response to stress and regulates the transcription of factors that contribute to the inflammatory SASP [36, 37]. As shown in Figs. 4A and B, levels of phosphorylated p65 (serine 536) were significantly higher in the glands from E-R72 compared to E-P72 mice, demonstrating differential activation of the NFκB pathway. In addition, glands from E-R72 mice had increased expression of genes that regulate acquisition of the secretory phenotype. Expression of *Pai1*, an angiogenic factor that also contributes to sustained cellular senescence [38], was significantly increased in glands of E-R72 compared to E-P72 mice (Fig. 4C). The matrix remodeling protease gelatinase-B (*Mmp9)* is associated with breast cancer risk [39], and *Mmp9*, but not *Mmp3*, was elevated in glands from R72 mice. Expression of the proinflammatory cytokines *Tnfα* and *Il6*, but *not Il8*, were also significantly increased in mammary glands of E-R72 mice compared to their E-P72 counterparts (Fig. 4C).

Inflammation also stimulates vascular dilation and increased capillary density as part of the immune response [40]. VEGFA is one of the best studied angiogenic factors, and variants have been associated with increased risk of a variety of cancers, including BrCa [41]. A significant increase in *Vegfa* expression was observed in the glands of E-R72 mice (Fig. 4C). Vascular density, assessed by immunohistochemical localization of the endothelial cell marker CD31 [42], was also significantly increased (Fig. 4D, E).

### Influx of proinflammatory macrophages in susceptible glands of E-R72 mice

**Fig. 5:**
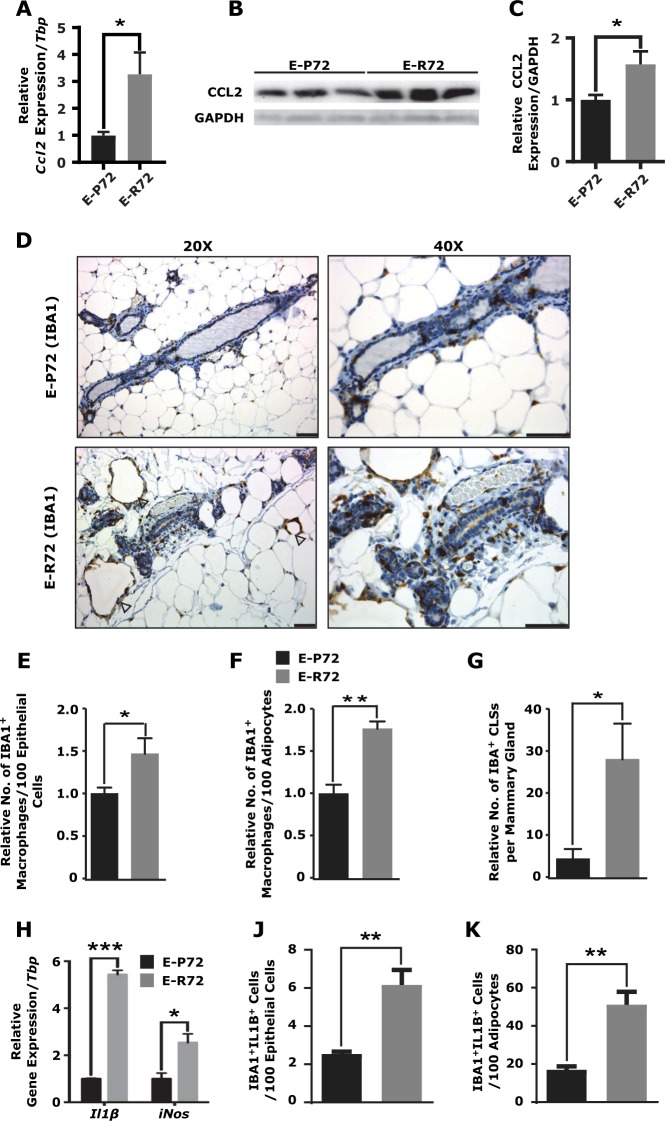

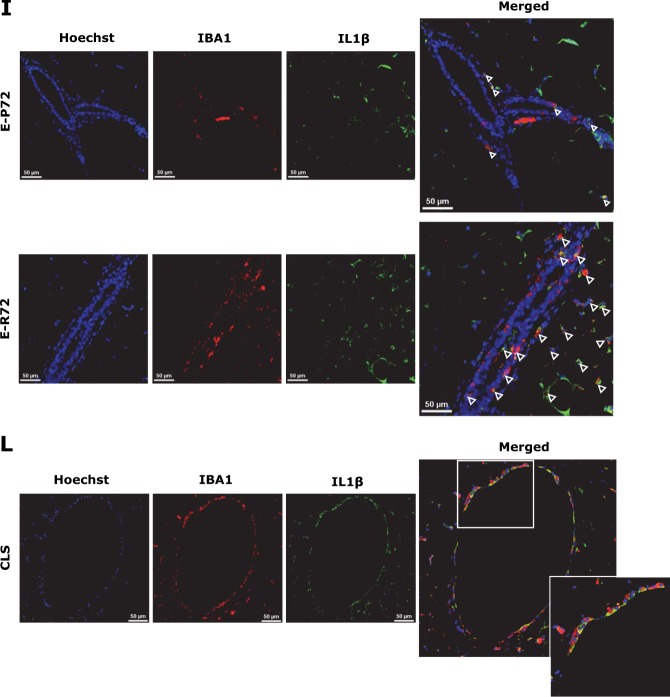
Influx of proinflammatory macrophages in susceptible glands of E-R72 mice. Comparisons are of mammary glands harvested from age-matched E-P72 and E-R72 mice. **A**. Quantitative RT-PCR of *Ccl2* normalized to *Tbp* (*n* = 9, *p < 0.05). **B**. Representative western blots of CCL2 and GAPDH. **C**. Densitometric quantitation of CCL2 protein, normalized to GAPDH (*n* = 4, *p < 0.05). **D**. Immunohistochemical localization of IBA1 (*n* = 5). Magnifications 20x and 40x, scale bar, 50 µm. Quantification of **E**. IBA1+ macrophages per 100 epithelial cells, **F**. IBA1+ macrophages per 100 adipocytes, and **G**. crown-like structures per mammary gland (*n* = 5, *p < 0.05, **p < 0.01). **H.** QPCR analysis of *Il1β* and *iNos*, normalized to *Tbp* (*n* = 4, *p < 0.05, ***p < 0.001). **I**. Indirect multiplex immunofluorescence images of macrophages. Hoechst dye (blue), IBA1 (red), IL1β (green), dual IBA1 and IL1β (yellow). Magnifications 20x and 40x, scale bar 50 µm. Arrowheads identify dual-stained IBA1+ IL1β+ macrophages. Quantification of IBA+ IL1β+ macrophages **J**. per 100 epithelial cells, and **K**. per 100 adipocytes, (*n* = 5, **p < 0.01). **L**. Indirect multiplex immunofluorescence image of macrophages forming a crown-like structure. Hoechst (blue), IBA1 (red), and IL1β (green). Magnification 40x, scale bar 50 µm. Inset: IBA1+ IL1β+ macrophages, white box identifies the magnified area. All values were standardized to the mean of E-P72 samples, and reported as mean ± SEM

The influx and persistence of proinflammatory macrophages are also critical indicators and contributors to chronic tissue inflammation [43]. CCL2 is a major driver of macrophage infiltration and has been shown to promote tumor progression in several cancer models [44]. Expression of this chemoattractant was increased in mammary glands of E-R72 compared to E-P72 animals, by 3.3 times at the message level (Fig. 5A), and 1.5 times at the protein level (Figs. 5B and C).

Macrophage influx was evaluated using the pan-macrophage marker, IBA1. Immunohistochemical localization of IBA1 revealed a significantly increased number of macrophages surrounding the ducts (Fig. 5D, E) and within the white adipose tissue (WAT) (Fig. 5D–F) of susceptible mammary glands from E-R72 compared to E-P72 mice. An increase in the number of crown-like structures (CLSs), formed when macrophages surround adipocytes, was also detected in glands from E-R72 animals compared to E-P72 glands (Fig. 5D–G). CLSs are often observed in chronically inflamed breast tissue from obese women, as well as in mammary tissues from obese mice [45, 46] and women with breast cancer [47].

Proinflammatory macrophages produce cytokines such as IL1β, and induce reactive nitrogen species (RNS) through upregulation of inducible nitric oxide synthase (iNOS) [48, 49]. Expression of *Il1β* and *iNos* were significantly upregulated in mammary glands of E-R72 mice (Fig. 5H). Dual immunofluorescent localization of IBA1 and IL1β revealed a significant increase of proinflammatory macrophages around mammary ducts (Fig. 5I and J) and adipocytes (Fig. 5I–K) in glands of E-R72 compared to E-P72 mice. As shown in Fig. 5L, macrophages in CLSs also expressed high levels of IL1β, providing further evidence of elevated and persistent inflammation in the susceptible glands of E-R72 compared to E-P72 mice.

### Increased tumor-associated macrophages (TAMs) and vascular density in mammary tumors from E-R72 mice

**Fig. 6:**
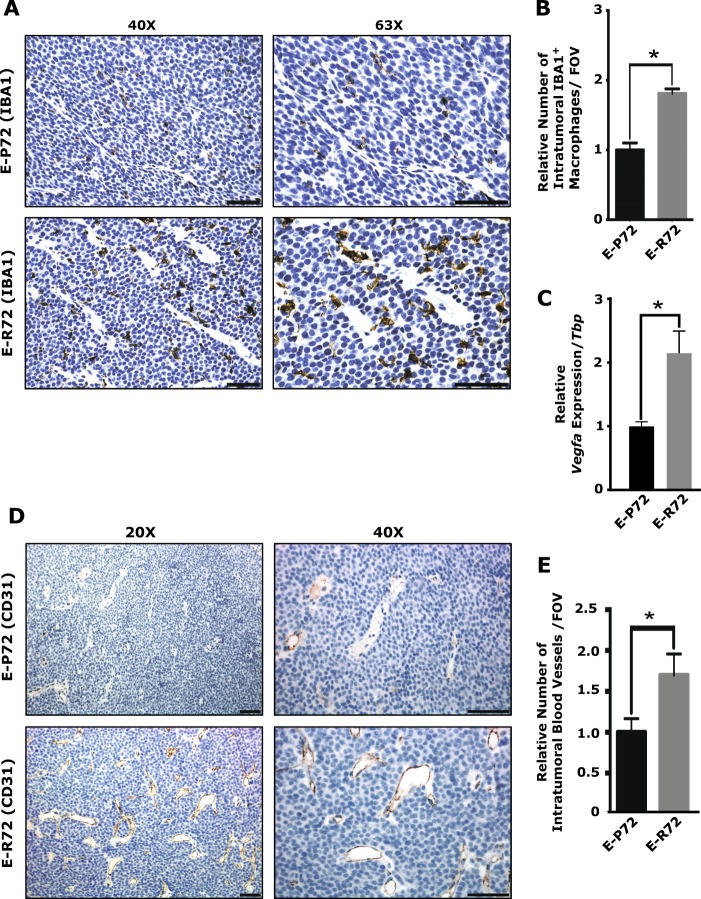
Increased tumor-associated macrophages (TAMs) and vascular density in mammary tumors from E-R72 mice. Comparisons are of mammary tumors harvested from E-P72 and E-R72 mice. **A**. Immunolocalization of IBA1. Magnifications 20x and 63x, scale bar, 50 µm. **B**. Quantification of IBA1+ macrophages per FOV (*n* = 4, *p < 0.05). **C**. QPCR analysis of *Vegfa*. Relative mRNA expression was normalized to *Tbp* (*n* = 5, *p < 0.05). **D**. CD31 immunolocalization. Magnifications 20x and 40x, scale bar, 50 µm. **E**. Quantification of CD31+ blood vessels per FOV (*n* = 5, *p < 0.05). All values were standardized to the mean of E-P72 samples, and reported as mean ± SEM

Recruitment of macrophages to primary tumor sites is essential for tumor progression, and inhibition of CCL2 significantly reduces macrophage infiltration and mammary tumor growth in MMTV-*PyMT* mice [50]. TAMs promote an immunosuppressive and angiogenic environment that further stimulates tumor growth and progression [51, 52]. Immunohistochemical analysis revealed significantly greater numbers of IBA1+ macrophages in mammary tumors from E-R72 compared to E-P72 animals (Figs 6A, B).

TAMs also secrete VEGFA, which promotes angiogenesis and supports tumor progression [49, 52]. As shown in Fig. 6C, *Vegfa* gene expression was elevated in tumors from E-R72 compared to E-P72 mice. Density of intratumoral blood vessels, assessed by the immunolocalization of CD31, was also elevated in mammary tumors from E-R72 animals (Fig. 6D, E). Together, these results show that influx of TAMs, along with elevated levels *Vegfa* and angiogenesis, contributed to the enhanced tumor progression in E-R72 mice.

### Enhanced binding of R72 to promoters of cell cycle arrest and inflammation genes

**Fig. 7:**
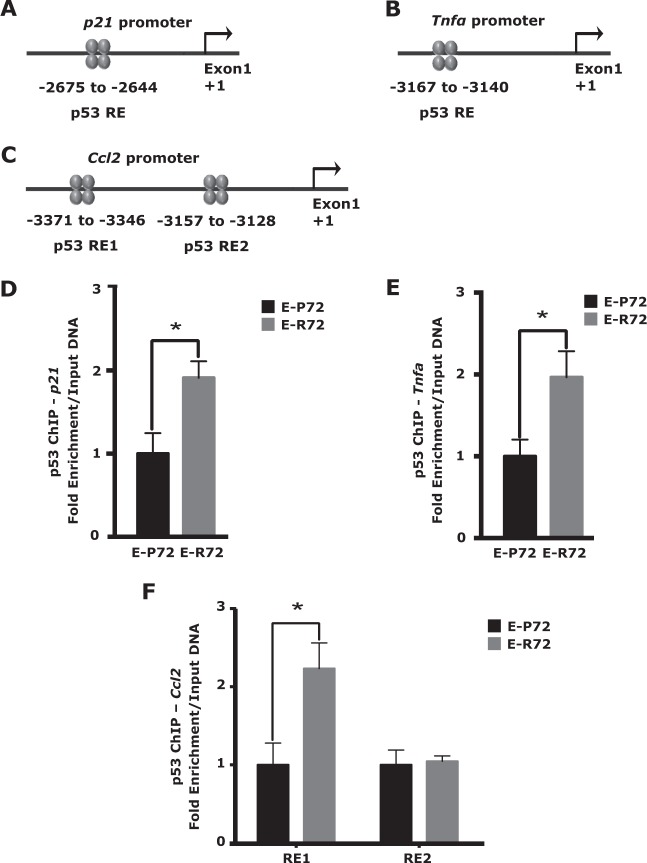
Enhanced binding of R72 to promoters of cell cycle arrest and inflammation genes. Location of p53 response elements (REs) in the distal promoters of **A**. *p21*, **B**. *Tnfα*, and **C**. *Ccl2* genes, relative to exon 1 (denoted as + 1). Comparisons are of mammary gland extracts from age-matched E-P72 and E-R72 mice. ChIP analysis of p53 binding to REs in the promoters of **D**. *p21*, **E**. *Tnfα*, and **F**. *Ccl2* genes. All values were standardized to the mean of E-P72 samples, and reported as mean ± SEM (*n* = 3, *p < 0.05)

P53 directly transactivates *Tnfα, Ccl2*, and *p21* by binding to canonical p53 response elements (REs) in their respective promoter regions [19, 53]. As discussed above, these genes are critical effectors of cellular senescence, SASP and inflammation, and their expression was significantly upregulated in susceptible mammary glands from E-R72 animals (Figs. 3C and 4C). In vivo ChIP- QPCR analysis was used to examine the association of the p53 variants with the REs of their target gene promoters. ChIP assays of extracts from susceptible mammary glands revealed a significantly greater enrichment of the R72 variant at the gene promoters of *p21* and *Tnfα* (Figs. 7A, B, D and E). For *Ccl2*, binding to RE 2 was similar in both genotypes, however, p53 binding to RE1 was significantly higher in E-R72 animals compared to E-P72 animals (Figs. 7C and F). These results indicate that the R72 variant had an increased affinity for specific REs, resulting in an increased ability to bind to and transactivate these critical genes involved in cellular senescence, macrophage recruitment, and chronic inflammation, ultimately leading to a protumorigenic tissue milieu.

## Discussion

This study examined the impact of the p53 codon 72 polymorphic variants on mammary tumorigenesis in a humanized (“knock-in”) mouse model. Results show that the codon 72 p53 polymorphisms differentially impacted cancer susceptibility and latency in both the DMBA-induced and MMTV-*Erbb2/Neu* mammary tumor models, representing two distinct tumor etiologies. Compared to the P72 variant, increased mammary tumor incidence and reduced latency were seen in mice homozygous for the R72 variant in both models.

Analysis of susceptible mammary glands revealed an increase in accumulation of senescent cells and SASP-mediated chronic inflammation in E-R72 glands compared to their E-P72 counterparts. Cell cycle regulators *p16*^INK4A^ and *p21*, which are effectors of senescence [28, 29], were also upregulated in mammary glands of E-R72 mice. R72 showed a greater affinity for the p53 RE in the distal portion of the *p21* promoter compared to P72, indicating a direct role of R72 in mediating accumulation of senescent cells in the tissue.

SASP in E-R72 mice was further stimulated by increased expression of *Pai1*, an effector of senescence [38]. Activation of NFκB, which initiates SASP in senescent cells, was also significantly elevated in mammary glands of E-R72 mice. NFκB pathway activation results in increased expression of proinflammatory cytokines that have been shown to induce a chronically inflamed tissue milieu [36]. Proinflammatory cytokines *Tnfα* and *Il6*, but not *Il8*, were upregulated in the chronically inflamed E-R72 glands. Our results also show that R72 played a direct role in increasing expression of *Tnfα*, since a significantly greater enrichment of the R72 variant of p53 was detected on the *Tnfα* promoter.

Increased influx of proinflammatory macrophages is also a hallmark of chronic inflammation [54]. In mammary glands of R72 mice, CCL2 was significantly elevated, and there was increased association of the R72 variant with the p53 RE in the distal promoter of the *Ccl2* gene. Results also revealed an increased influx of IBA1+ IL1β+ proinflammatory macrophages into the mammary glands of E-R72 mice, which contributed to a chronically inflamed tissue milieu. During chronic inflammation, proinflammatory macrophages produce reactive nitrogen species (RNS) through induction of iNOS, resulting in mutagenic oxidative DNA damage, which contributes to genomic instability [43]. In colorectal cancer, RNS-initiated DNA damage accelerates loss of *Apc* and enhances tumor development [55]. Our results also show that *iNos* expression was increased in the susceptible mammary glands of E-R72 mice, indicating that production of RNS enhanced mutagenic transformation of mammary epithelium.

Another contributor to the protumorigenic milieu is angiogenesis, which is essential for maintenance of chronic inflammation [40]. MMP9 increases the bioavailability of VEGFA, which induces angiogenesis and vasodilation [56]. Both *Vegfa* and *Mmp9* were significantly elevated in mammary glands from E-R72 compared to E-P72 mice, along with an increase in CD31+ blood vessels. Increased angiogenesis is seen in susceptible mammary tissue in mouse models that overexpress *Cox2* or *Ccl2* [57, 58], and reduction of angiogenesis by genetic ablation of *Cox2* significantly reduces MMTV-*Erbb2*/*Neu*-induced mammary tumor incidence [59].

The proinflammatory activities of R72 have also been reported in the Hupki model, particularly when mice are maintained on a high-fat diet. The R72 variant more effectively transactivates *Tnfα* and *Ccl2* genes compared to P72, inducing inflammation in the liver and WAT. This effect is accompanied by an increased proportion of senescent islet cells in the pancreas of R72 compared to P72 mice. In the liver of these Hupki mice, expression of *Tnfα* and *Npc1l1* genes is higher in R72 than P72 mice, where they induce inflammation and increase cholesterol absorption, respectively, predisposing R72 animals to obesity and insulin resistance [19]. These results are in agreement with our findings that the R72 variant more effectively induced inflammation and associated disease progression in the mammary gland.

Interestingly, a recent human epidemiological study demonstrates an association of the R72 variant with inflammatory irritable bowel disease (IBD), which is an important risk factor for colorectal cancer (CRC) [60]. Consistent with this, positive association studies from Iran [61], Greece [62], and Argentina [63] have shown that R72 predicts increased susceptibility to CRC. On the other hand, studies conducted in Turkey [64] and Malaysia [65] have found that P72 is associated with increased risk, indicating a possible role for unique and variable gene-environment interactions, including diet, obesity, and other exposures that affect chronic inflammation.

The association of the p53 codon 72 SNP with BrCa risk is complex. Several studies report that mutated R72 is preferentially retained in breast, colorectal, and head and neck cancers [21, 22, 66], where it reduces apoptosis, and contributes to therapeutic resistance and tumor progression [67]. The retained, mutated R72 allele is also associated with decreased disease-free interval and overall survival in BrCa [21]. Epidemiological studies have found that the R72 variant is associated with BrCa risk and reduced overall survival [11], and some of the strongest associations are in cohorts of Asian patients [20, 24]. Other studies, conducted in predominantly White patient populations, have found that the P72 variant is associated with increased BrCa risk [68]. Taken together, these results indicate that further studies with diverse patient populations are needed to clarify the role of the 72 SNP in BrCa susceptibility globally.

Our studies in a humanized mouse model demonstrated that the p53 codon 72 polymorphic variants differentially affected susceptibility and kinetics of mammary tumorigenesis, and provided evidence for a mechanistic link between R72 and chronic, protumorigenic tissue inflammation. These studies also illustrate that single-nucleotide polymorphisms can have a profound effect on tumorigenesis in vivo. Given the adverse role of chronic inflammation in tumorigenesis, codon 72 variants may predict, at least in part, breast cancer susceptibility, disease progression and/or treatment outcome.

## Materials and methods

### Maintenance and genotyping of mice

Transgenic mice used for these studies were on an FVB background and were homozygous for the proline (P72) or arginine variant (R72). PCR-based genotyping of mice was performed as described previously [17]. Genotypes were verified by a custom quantitative reverse transcription PCR (RT-PCR) SNP allelic discrimination assay (AH89RLW, Thermo Fisher Scientific, Waltham, MA) using an ABI7900HT real-time PCR instrument (Applied Biosystems, Inc., CA). All study mice were generated from genotype-confirmed parents.

Breeders were maintained on standard chow ad libitum, and tumorigenesis study animals were switched to AIN76A (F1515, Bio-Serv, NJ) diet after weaning. Mice were provided free access to drinking water and were housed in Association for Assessment and Accreditation of Laboratory Animal Care (AAALAC)-certified, temperature- and humidity-controlled facilities, with a 12-h light/12-h dark cycle. All procedures were performed according to protocols approved by the Institutional Animal Care and Use Committee at Texas A&M University, College Station, TX.

### Tumorigenesis experiments

#### DMBA treatment of mice

Starting at 16 weeks of age, 39 P72 and 40 R72 mice were administered 1 mg of 7, 12-dimethylbenz[a]anthracene (DMBA) dissolved in 100 μL corn oil, once a week for 6 weeks by oral gavage. Similarly, another cohort of 16-week-old P72 (*n* = 19) and R72 (*n* = 21) animals were administered 100 µL of corn oil vehicle once a week for six weeks by oral gavage.

#### Generation of MMTV-*Erbb2*/*Neu* P72R variant mice

FVB/N-Tg(MMTVneu)202Mul/J mice were purchased from Jackson Laboratories (Bar Harbor, ME) and bred with *p53* polymorphic mice to generate bigenic mice that were hemizygous for *Erbb2* and homozygous for the *p53* variants. *Erbb2* copy number was determined by quantitative RT-PCR according to the Jackson Laboratories protocol (https://www2.jax.org/protocolsdb/f?p = 116:5:0::NO:5:P5_MASTER_PROTOCOL_ID,P5_JRS_CODE:30062,002376). A total of 54 E-P72 and 56 E-R72 mice were used for this study.

Large cohorts of non-randomly assigned animals were used in tumorigenesis studies to ensure adequate statistical power. Animals were monitored daily for health status and tumors in non-blinding manner. Mice were palpated twice weekly throughout the study and sacrificed when the tumor reached 1.5 cm in any direction. Mice were sacrificed by CO_2_ asphyxiation, mammary tumors were measured and tissues harvested for later analyses. Tumor histology was evaluated by two independent pathologists.

#### Protein extraction and western blots

Protein from 6-month-old E-P72 and E-R72 mammary glands was extracted from using boiling 2x Laemmli sample buffer, and concentrations were determined with the BCA protein assay kit (Thermo Fisher Scientific, Waltham, MA) according to the manufacturer’s protocol. Equal amounts of total protein were resolved by sodium dodecyl sulphate polyacrylamide gel electrophoresis and transferred to polyvinylidene fluoride (PVDF) membranes. Membranes were probed with following primary antibodies: from Cell Signaling Technologies (CST): CCL2 (1:1000, #2029) GAPDH (1:5000, #2118), Phospho-RB (1:1000, #8516), Phospho-p65 (Ser536) (1:1000, #3033), TNFα (1:1000, #11948), from Santa Cruz Technologies: p53 (1:500, sc-6243) and p21 (1:500, sc-397).

All blots (except GAPDH) were incubated with goat anti-rabbit HRP-conjugated secondary antibody (1:2500, CST, #7074) and developed using ECL Prime reagents (GE Healthcare, IL). Images were captured using a FluorChem M imager and quantified with AlphaView software (ProteinSimple, CA). GAPDH blots were incubated with iRDye800CW secondary antibody (1:5000, LI-COR Biosciences 925–3211) and developed and imaged using the Odyssey Li-COR system (LI-COR Biotechnology, NE).

#### RNA extraction and quantitative RT-PCR

Mammary glands harvested from 6-month-old E-P72 and E-R72 animals were homogenized with a Kinematica Polytron^™^ bench-top homogenizer and RNA was extracted with the Maxwell^®^ 16 LEV simplyRNA Tissue Kit, according to the SimplyRNA tissue protocol (Promega Corporation, WI). Samples were randomly checked for RNA quality using the 2200 TapeStation instrument, following the manufacturer’s protocol (Agilent Technologies, Inc., CA). Total RNA was then reverse transcribed as previously described [69]. Quantitative RT-PCR analyses were performed on an ABI7900HT real-time PCR instrument. TaqMan gene expression assays were used for *p53* (Mm01731290_g1) and *p21* (Mm04205640_g1, Thermo Fisher Scientific, Waltham, MA). Previously published quantitative RT-PCR primers for SYBR green reactions of *Erbb2*, *Il6*, *Il8*, *p16*^INK4a^, *Pai1, Tnfα, Ccl2, Vegfa*, *Mmp3*, *Mmp9*, *Il1β* and *iNos* were used to determine target gene expression [19, 49, 70–74]. Each sample was normalized to corresponding TATA box binding protein (*Tbp*) gene expression, using a TaqMan assay (Mm00446973_m1, Thermo Fisher Scientific, Waltham, MA) or published primers [71], run in triplicate, and quantified using the ΔΔ*C*_t_ method.

#### Chromatin immunoprecipitation (ChIP)

Chromatin from mammary glands of 6-month-old E-P72 and E-R72 mice was isolated as described [75]. In brief, approximately 300 mg of tissue were minced in cold PBS and cross-linked in 1% freshly-made paraformaldehyde-PBS for 10 min. Cross-linking was quenched by adding glycine to a final concentration of 125 mM, and homogenized with a Dounce homogenizer in cold cell lysis buffer (10 mM Tris-Cl, pH 8.0, 10 mM NaCl, 3 mM MgCl_2_, 1% NP-40) supplemented with protease inhibitors (Roche, #04693159001) to generate a single-cell suspension. Cells were incubated on ice, and centrifuged at 1000×*g* for 10 min at 4 °C to pellet nuclei. The pellet was then resuspended in nuclear lysis buffer. Chromatin was sheared by sonication to an average size of 200 to 1000 bp using a Bioruptor (Diagenode, NJ), and insoluble debris was removed by centrifugation. Immunoprecipitation was performed using 10 µg of p53 (FL393) antibody (sc-6243). Primers 5ʹ AAAATCGGAGCTCAGCAGGCCT 3ʹ (forward) and 5ʹ ATCAGGTCTCCACCACCCTGC 3ʹ (reverse) were used for ChIP analysis of the p53 RE in *p21* promoter. The quantitative RT-PCR primers used to measure *Tnfα* and *Ccl2* p53 REs were previously published [19].

#### Sudan Black B (SBB) staining for senescence detection

SBB staining was performed on sections of mammary glands from 6-month-old E-P72 and E-R72 animals by the Texas A&M College of Veterinary Medicine histology core laboratory, using the Sudan Black B Histochemical Stain Kit, following the manufacturer’s protocol (American MasterTech, KTSBBPT).

#### Immunohistochemistry of Ki67, CD31, IBA1, and CC3

IHC was performed by the Immunohistochemistry Core Laboratory at Texas A&M or the Pathology Core Laboratory at Baylor College of Medicine using standard protocols. Briefly, mammary sections from 6-month-old E-P72 and E-R72 FFPE mice were deparaffinized in xylene (VWR, MK866802), rehydrated in graded ethanols (100%–95%–70%) and distilled water. Antigen retrieval was performed in 0.1 M Tris-HCl, pH 9.0 for 15 min. Endogenous peroxidase activity was quenched with 3% hydrogen peroxide (VWR, BDH7540-2). Sections were blocked in 10% bovine serum albumin in Phosphate Buffered Saline with Tween 20 (PBST). Tissue sections were incubated for 45 min with primary antibody, and 1 h with HRP-conjugated anti-rabbit IgG secondary antibody (1:500, Abcam, ab6721). Color was developed by adding the substrate and chromogen 3,3**′**-diaminobenzidine (DAKO). The primary antibodies used in IHC staining were Ki67 (1:500, Abcam, ab15580), CC3 (1:200, CST, 9664), IBA1 (1:500, WAKO, 019–19741), and CD31 (1:200, Abcam, ab28364). Sections were counterstained with hematoxylin (VWR, RC353032) and visualized with an automated upright microscope (LeicaDM5500B, Leica Biosystem, Germany). Quantification of images was performed using the ImmunoRatio plug-in, Fiji software [76] or blinded, manual counting of at least six high power fields.

#### Multiplex indirect immunofluorescence

FFPE mammary gland sections from 6-month-old E-P72 and E-R72 animals were deparaffinized in xylene (VWR, MK866802) and rehydrated in graded ethanols (100%–95%–70%) and then 1x PBS. Antigen retrieval was performed by boiling in sodium citrate solution, pH 6.0 for 10 min. After washing, sections were blocked with 10% horse serum in PBST. Tissue sections were incubated overnight at 4 °C with primary antibodies, washed, and incubated with Alexa dye-conjugated anti-goat/anti-rabbit IgG for 1 h at room temperature (RT). Primary antibodies were: IBA1 (1:100, WAKO, 019–19741) and IL1β (1:50, R&D Systems, AF-401-NA). Sections were visualized using an A1R HD confocal microscope (NIKON Instruments Inc., NY). Images were captured using NIS elements software and blindly quantified in six high power fields.

### Statistical analysis

Log-rank test for trend was performed to statistically compare the Kaplan–Meier curves. Fisher’s exact test and Chi-square tests were performed to compare tumor incidence. When animals were sacrificed before the end of study (at 1 year of age), due to causes other than a mammary tumor, they were censored (removed) from the Kaplan–Meier curves on the day of sacrifice. However, the mammary tumor incidence graphs include only animals that had a histologically confirmed mammary tumor, or that survived tumor-free until the end of study. Unpaired two-sided Student’s *t*-test, assuming more conservative unequal variance, was used to compare tumor growth rates, quantitative RT-PCR, IHC, and densitometry analyses. All statistical analyses were performed using GraphPad Prism software (version 6). A *p*-value < 0.05 was considered statistically significant (*p < 0.05, **p < 0.01 and ***p < 0.001).
